# Multidisciplinary oral rehabilitation of an adolescent suffering from juvenile Gorlin-Goltz syndrome – a case report

**DOI:** 10.1186/s13005-019-0189-5

**Published:** 2019-02-08

**Authors:** Manfred Nilius, Jürgen Kohlhase, Johann Lorenzen, Günter Lauer, Matthias C. Schulz

**Affiliations:** 1 Niliusklinik Dortmund, Londoner Bogen 6, 44269 Dortmund, Germany; 2 Humangenetik Freiburg GmbH, Heinrich-von-Stephan-Str. 5, D-79100 Freiburg, Germany; 3 Department of Pathology, Klinikum Dortmund gGmbH, Beurhausstraße 40, D-44123 Dortmund, Germany; 4 Department of Oral and Maxillofacial Surgery, University Hospital “Carl Gustav Carus”, Technische Universität Dresden, Fetscherstr. 74, D-01307 Dresden, Germany

**Keywords:** Bone graft, Dental implant, Distraction osteogenesis, Keratocystic odontogenic tumor

## Abstract

**Background:**

The Gorlin-Goltz syndrome is an autosomal dominant disorder characterized by keratocystic odontogenic tumors in the jaws, multiple basal cell carcinomas and skeletal abnormities. Frequently, the manifestation of the syndrome occurs in the adolescent years.

**Case presentation:**

An 11-year-old boy was referred to our clinic due to the persistence of the lower deciduous molars. The further diagnosis revealed bilateral keratocystic odontogenic tumors in the region of teeth 33 and 45 representing a symptom of a Gorlin-Goltz syndrome. This case of the oral rehabilitation of an adolescent with bilateral keratocystic odontogenic tumors shows the approach of a multidisciplinary treatment concept including the following elements: Enucleation and bone defect augmentation using a prefabricated bone graft; distraction osteogenesis to extend the graft-block vertically after cessation of growth; accompanying orthodontic treatment, guided implant placement and prosthetic rehabilitation. Six months after implant insertion, a new keratocystic odontogenic tumor in the basal part of the left sinus maxillaris had to be removed combined with the closure of the oroantral fistula. During the follow-up period of 18 months in semi-annual intervals, the patient showed no sign of pathology.

**Conclusion:**

In the presented case could be shown that distraction osteogenesis of prefabricated bone blocks is possible. With a multidisciplinary approach in a long-term treatment a sufficient oral rehabilitation of the patient suffering from extended keratocystic odontogenic tumors was possible.

## Background

The Gorlin-Goltz syndrome, also referred as Nevoid Basal Cell Carcinoma Syndrome (NBCCS) or Basal Cell Nevus Syndrome (BCNS) was first described by Gorlin et al. in 1960 although it was known for decades before [1]. It is an autosomal dominant disorder characterized by KeratoCystic Odontogenic Tumors (KCOT) in the jaws, multiple basal cell carcinomas and skeletal abnormities [2–7]. As epidemiological studies are rare the prevalence differs from 1:50,000 to 1:150,000. Frequently, the manifestation of the syndrome occurs in the juvenile [3, 5, 6].

In 2005, the classification of the World Health Organization (WHO) was changed from the former term odontogenic keratocyst (OCT) to keratocystic odontogenic tumors as a subgroup of neoplasms deriving from the odontogenic epithelium [8]. The keratocystic odontogenic tumors develop in the dental laminal epithelium caused by a malfunctioning regulation of growth factors, cytokines, cell cycle and signal transduction [9–11]. Furthermore, polymorphisms of regulatory genes like IL-1α might play a role, likewise [12]. Keratocystic odontogenic tumors consist of multilayered squamous epithelium and tend to form focal hyperplasia as well as secondary cysts in the adjacent connective tissue [13, 14]. In an early stage, KCOT radiographically look like follicular residual cysts presenting as sharply marked unilocular or multilocular translucent areas potentially masking the diagnosis [15]. The prevalence of keratocystic odontogenic tumors is three times higher in the mandible compared to the maxilla. The region which is mainly affected is the area distal of the third molar [7, 13, 16].

According to recent findings, the NBCCS and, keratocystic odontogenic tumors, likewise, were described to not only appear in an inherited way but also sporadically following mutation of the PTCH1 or PTCH2 [9q22.3] gene [14, 17–20]. Keratocystic odontogenic tumors associated with Gorlin-Goltz syndrome show a high proliferation rate [5, 9]. Without sufficient therapy they might lead to aggressive destruction of jaw bone and thus, leading to facial deformation or asymmetry. The aggressive pattern of growths and the tendency to recurrence require a thoroughly performed therapy depending on the histologic subclassification [3, 14, 21–24]. Currently, the enucleation of keratocystic odontogenic tumors is recommended in combined with curettage or with ostectomy as the least invasive therapy [3, 14, 25, 26]. It is crucial completely remove the KCOT in order to avoid the release of activated cells in deeper tissue layers. Secondary cysts should be eliminated, likewise and ought to be analyzed pathologically. Considering the surgical management of extended bone defects in the adolescent two issues are challenging for the clinician: the rehabilitation with autologous bone is considered as golden standard [27]. However, a limited amount of autogenous bone and the donor site morbidity are factors limiting the use of autogenous bone [28]. Furthermore, the age of the patient and the planned therapy of oral rehabilitation have to be considered. The placement of dental implants during the growths is currently an issue of discussion [29]. Thus, an individual treatment plan considering the age of the patient, the extend of the defects and the planned oral rehabilitation has to be created. The presented case is describing the complex interdisciplinary long-term treatment of an adolescent patient suffering from Gorlin-Goltz syndrome. A potential approach to sufficient oral rehabilitation of such patients is reported.

## Case presentation

### Medical history and initial findings

An 11-year-old boy was referred to our clinic for further diagnostic due to the persistence of the lower deciduous molars. The family history was without pathological findings. The clinical extra-oral examination revealed a kyphoscoliosis accompanied by a pectus excavatum with an age-appropriate habitus. Additionally, a myopia with moderate hypertelorism was obvious.

The intra-oral examination showed a mixed dentition at the beginning of the second dentition phase. The following deciduous teeth were found to be in situ: 53, 55, 63–65, 73–75, 84 and 85. Furthermore, the permanent teeth 14, 16, 12–22, 26, 36, 32–43 and 46 were erupted. In the vestibular region of teeth 43 and 84 an indolent, solid, smooth-margined swelling with a diameter of 20 mm was obvious.

**Fig. 1:**
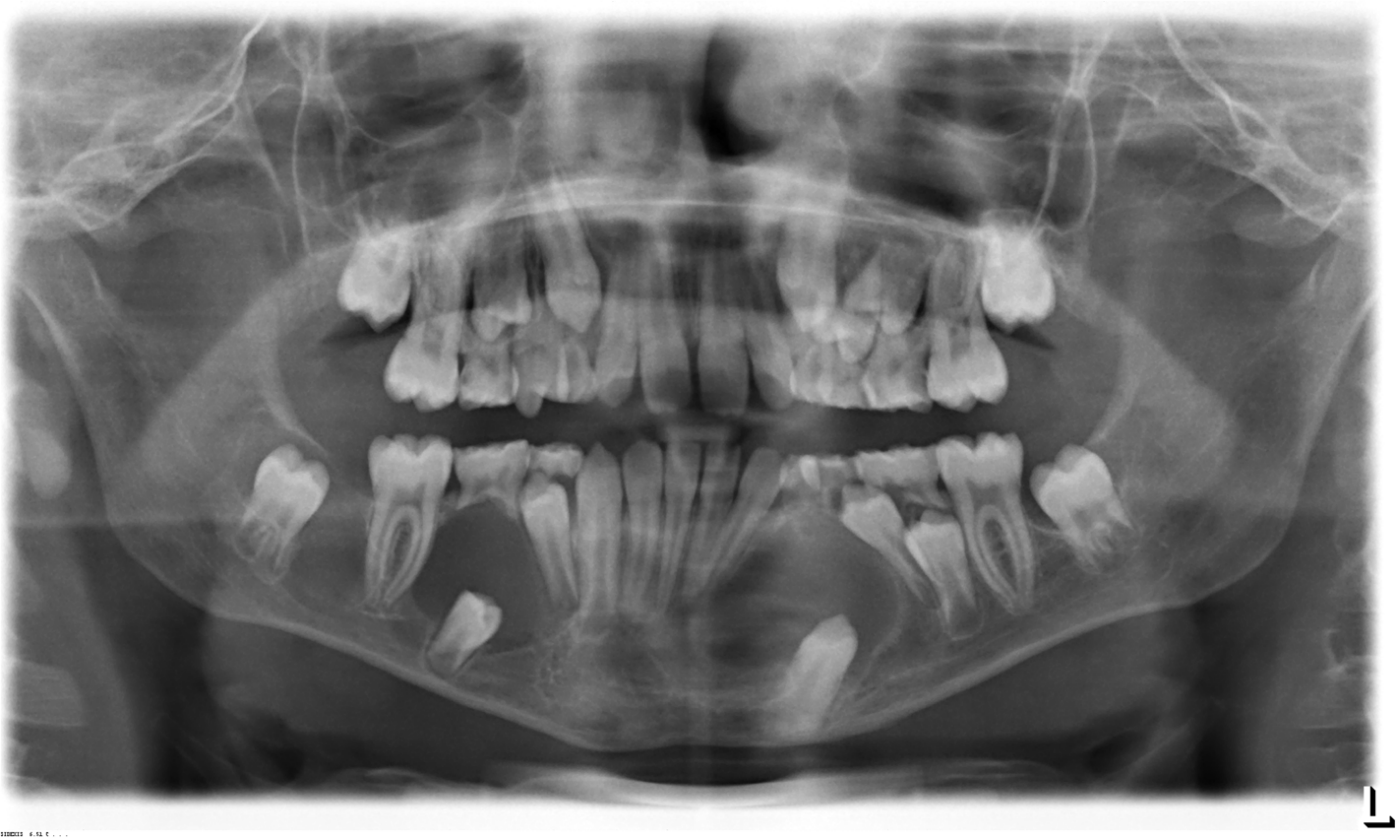
Initial orthopantomogram (OPT) at the age of 11 years showing the extended pericoronar osteolyses of the teeth 33 and 45 which were retained at the base of the mandible. The adjacent teeth (32, 34, 44) seem to be dislocated by the cystic lesions. In the area between teeth 46 and 47, a diffuse osteolysis confluent with a peri-coronar osteolysis around the retained 47 is obvious. Furthermore, root resorptions at the teeth 73 and 85 are visible

The orthopantomogram (OPT) showed a retention of the teeth 33 and 45 at the base of the mandible. In the peri-coronal area of both teeth enlarged radiolucencies with a clearly visible margin in region 33 and 45 were obvious. Additionally, in the area between teeth 46 and 47, a diffuse osteolysis confluent with a peri-coronar osteolysis around the retained 47 was visible. Furthermore, the teeth 35, 34 and 44 were angulated disto-mesially and root resorptions at the teeth 73 and 85 were observed. (Fig. 1).

### Diagnostic work-up

In order to clarify the clinical and radiographic findings an incisional biopsy was taken from region 33 and 45 in local anesthesia. The histological examination proved the a keratocystic odontogenic tumor in both regions. Due to the diagnosis of bilateral KCOT the clinical suspicion of a NBCCS (Gorlin-Goltz syndrome) arose. Thus, multi-disciplinary consultations including radiology of the head-neck region, a dermatological screening, ophthalmological and orthopedic examinations were initiated. Furthermore, a genetic evaluation of the family including the entire generation of grandparents based on a sequencing of all PTCH1 exons and their intron-exon boundaries was performed. The genetic screening revealed a spontaneous mutation of the PTCH1 gene in the mother. Thus, the initial suspicion of a Gorlin-Goltz syndrome could be confirmed. However, the mother’s clinical findings were without pathology so that she can be considered as a carrier without any clinical gene expression.

### Treatment plan

Due to the genetic aberration revealed by the blood test a genetic counseling was recommended to the family. Considering the pathological findings, an interdisciplinary treatment plan for the adolescent patient was created. In order to find a satisfying treatment for the patient the options of marsupialization and enucleation of the cystic lesions were discussed with the patient and his family. Due to the long treatment period associated with the marsupialization enucleation of the lesions with simultaneous augmentation of the resulting defects was preferred.

1. Follow-up of the physical screenings focusing on dermal and ophthalmic anomalies on a regular three-month interval. Radiological follow-up semi-annually focusing on osteolytic lesions in the jaw.
2. Enucleation of the KCOTs with simultaneous bone grafting.
3. Orthodontic treatment to level the dental arches and to maintain space for implant supported oral rehabilitation in region of 33 and the right mandible.
4. Insertion of dental implants and prosthetic restoration after the cessation of the physical growth.

### Enucleation of the KCOT with simultaneous bone grafting

**Fig. 2:**
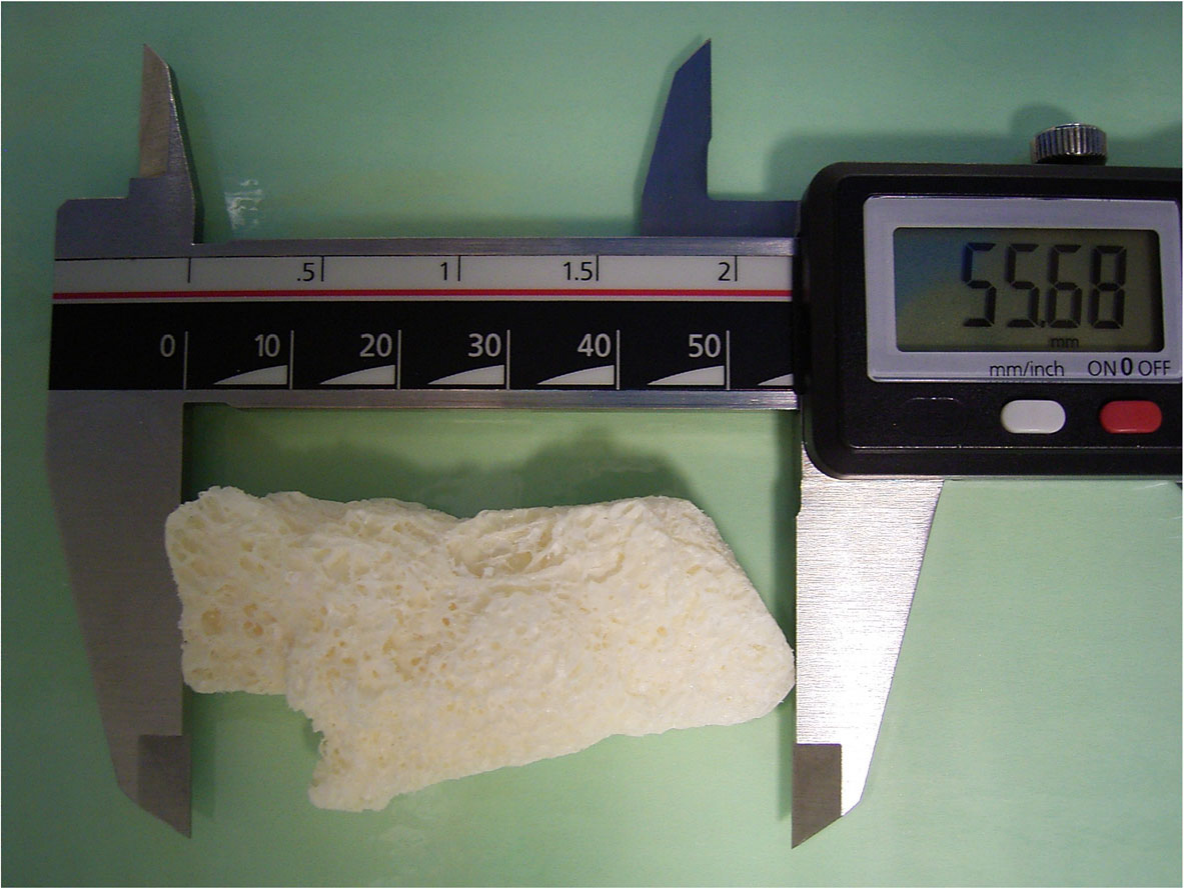
Custom-made bone block prior to insertion in the right mandible. The mesio-distal extension was 55 mm

**Fig. 3:**
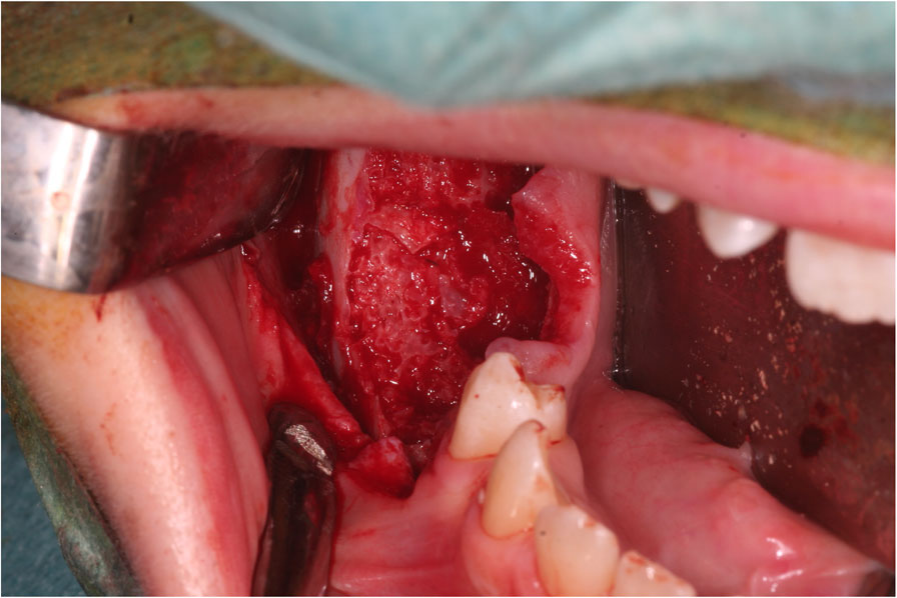
The custom-made bone block in situ

In order to estimate the extent of the bony defects remaining from the KCOTs, a cone beam computed tomography (CBCT, 3D-Exam, KaVo Dental GmbH, Biberach, Germany) was performed preoperatively. The bony defect in region 33 measured about 32 × 28 mm and the defect in region 45 was approximately 27 × 24 mm. The lesion in the right mandible showed a length of approximately 60 mm and a height and width of approximately 20 mm. Based on the data obtained from the CBCT, a model was created. Subsequently, a CAD/CAM milled, allogenic bone block was produced for the defect resulting from the enucleation in the right mandible (Tutoplast® Spongiosa, Tutodent, Neunkirchen a. B., Germany). The customized bone block is shown in Fig. 2. The cystectomy including the removal of the teeth 33, 45, 46 and 47 was performed in general anesthesia at the age of 12 years. It was possible to achieve an enucleation in toto that histologically confirmed a KCOT by instantaneous section diagnosis. In order to preserve the inferior alveolar nerve ostectomy or curettage were not carried out. Simultaneously, the reconstruction of the defects in region 33 and 45 were performed using preformed allogenic spongiosa blocks (Tutoplast® Spongiosa, Tutodent, Neunkirchen a. B., Germany). In the right mandible, the customized spongiosa block was inserted (Fig. 3). The wound closure was achieved using a porcine pericardium membrane (Botiss Jason® membrane, Straumann GmbH, Freiburg, Germany) and absorbable sutures (Vicryl 3–0, Ethicon Deutschland, Johnson & Johnson Medical GmbH, Norderstedt, Germany). The extraction of the deciduous teeth 63 and 65 and a peri-coronal cystectomy at tooth 23 were carried out in the same session. Again, instantaneous section diagnosis revealed a KCOT around tooth 23. As the KCOT was only located peri-coronally and tooth 23 was retained in a vertical position closed to eruption the preservation of 23 was intended. Thus, a bracket for forced orthodontic movement was attached to the tooth.

In order to prevent mesial movement of the teeth in the left mandible, the orthodontic treatment was initiated directly postoperatively. This involved the fine adjustment of the dental arches with orthodontic movement of tooth 23 into the intended position. Furthermore, the angulated teeth 32, 34 and 44 were erected. Thus, the cuspid space in region 33 was kept open and the remaining front teeth could be stabilized in order to allow insertion of dental implants in the right posterior mandible.

### Bone augmentation in the adolescent

In order to estimate the skeletal growth reserves a hand radiograph was performed at the age of 15 years. The method according to Björk is routinely used by pediatricians to determine the individual growth development and degree of maturation [30, 31]. This yielded a Ru-stage which implied the end of the physical growth according to ossification of the radius [32]. At this age, the patient’s height was already about 10 cm more than his parents’. The consultation of a pediatrician assumed the end of the growth period. Thus, it was decided to start the preparation of the implant placement.

**Fig. 4:**
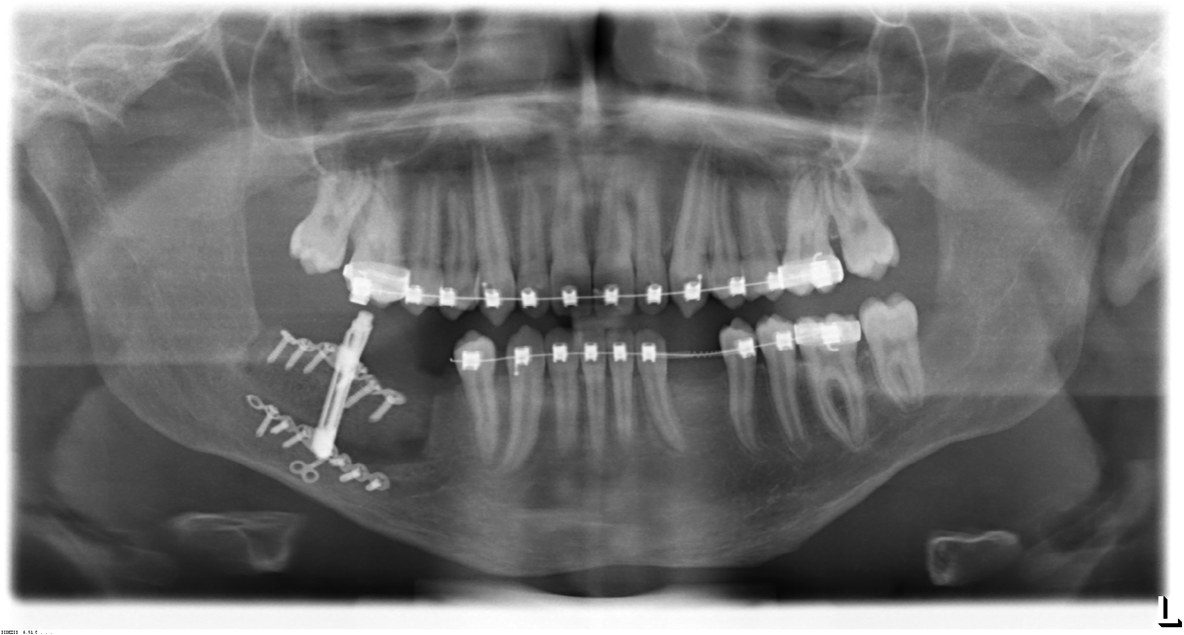
Orthopantomogram after insertion of the distractor and distraction to the maximal height. Subsequently, the consolidation phase followed

**Fig. 5:**
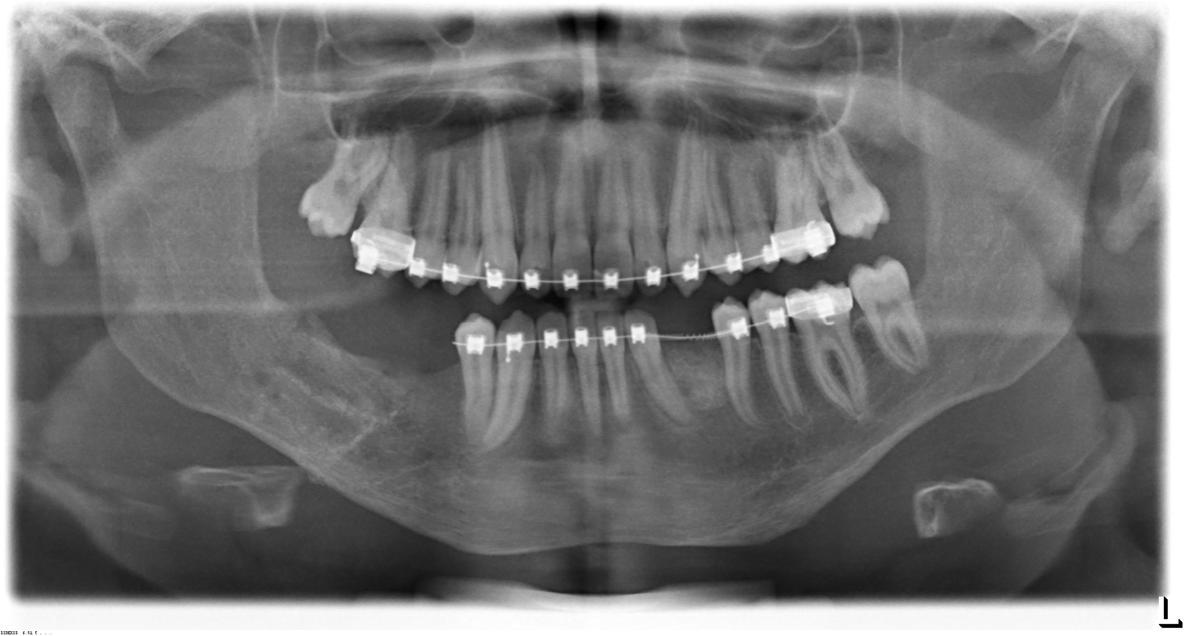
Orthopantomogram after finalization of the consolidation phase. A sufficient osteogenesis in the distracted area is achieved

For the detailed analysis of the bone volume, a CBCT was performed. The findings revealed an insufficient crestal amount of bone in region of tooth 33. Furthermore, a vertical deficit of about 8 mm in the right posterior mandible in regio 46 and 47 was obvious while the bone width was 12.9 mm. Only 9 mm of bone cranial of the mandibular canal could be measured providing an inappropriate crown to root relation. In general anesthesia, a lateral bone augmentation using xenogenic bone substitute (Cerabone®, Botiss GmbH, Zossen, Germany) was performed in region of tooth 33. Additionally, in order to overcome the vertical gap in the right mandible, the allogenic bone graft in region 45–48 was augmented by a discontinuous distraction osteogenesis (Vertical Alveolar Distraction, Type Cologne, Gebrüder Martin & Co KG, Tuttlingen, Germany). The healing was uneventful without any dehiscence or signs of inflammation. Subsequently, the distraction phase was set up for 18 days followed by a consolidation phase of 6 months (Fig. 4). Thus, a bone height of 15 mm cranial of the mandibular canal was achieved while the bone width decreased slightly to 11.5 mm which, however, could considered sufficient for the insertion of dental implants (Fig. 5).

### Implant placement and prosthetic restauration

**Fig. 6:**
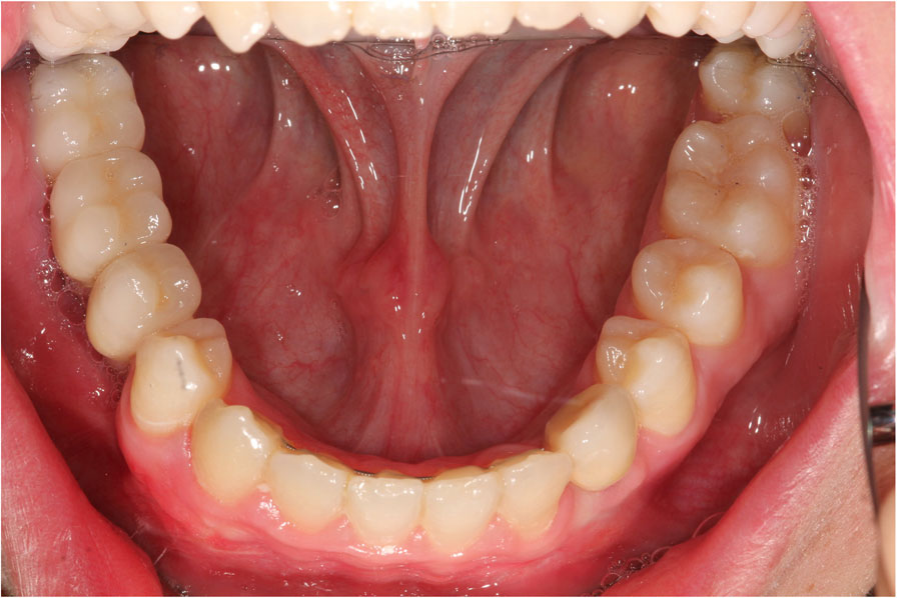
The clinical situation after incorporation of the final prosthetic restorations

The planning of the implant position was performed using the three-dimensional guided protocol of the NobelGuide® (NobelBiocare Deutschland GmbH, Cologne, Germany). In order to obtain samples for the histological examination of the augmented bone block a cylindrical trephine core of 3.5 mm diameter was harvested from region 45, 46 and 47. The samples were embedded in paraffin and stained using hematoxylin eosin. The histological examination revealed vital spongy bone trabeculae along avital bone tissue with evidence of increased remodeling. The implant insertion in areas 33, 45, 46 and 47 was performed according to the manufacturer’s protocol. Subsequently, four implants (SIC invent Deutschland GmbH, Göttingen, Germany) were placed in the regions of 33 and 45 (4.0 × 11.5 mm) and in regions of 46 and 47 (5.0 × 11.5 mm). The implants were allowed to heal for 6 months. During the healing period of the implants, the orthodontic fine adjustment was carried out. After 6 months, the implants were uncovered and provided with provisional crowns. An adaptation phase of another 6 months was allowed before incorporation of the final prosthetic restorations (Fig. 6). The porcelain fused to base metal crowns were fixed on custom made abutments. At this stage of the treatment, the patient was 18 years old (Table 1).

**Table 1:** Medical History Timeline

08/2006	first presentation due to persistence of lower deciduous molars
03/2007	suspicion of lesions in region 23 and right mandible
02/2008	genetic counseling
04/2008	removal of KCOTs in the mandible, removal of teeth 33, 45, 46, 47 and simultaneous bone augmentation
07/2009	insertion of distractor
11/2009	removal of distractor
02/2010	planning the implant insertion using NobelGuide®
03/2010	implant insertion
09/2010	uncovering of the implants and provisional fixed restorations
03/2011	definitive prosthetic restorations
07/2011	removal of germ 18, tooth 17 and a KCOT in right posterior maxilla
06/2014	removal of germ 28 and a KCOT in left posterior maxilla

### Outcome and follow-up period

Regarding the histologically proven KCOTs a three-months radiological control interval was set up during the treatment period until the eruption of the last permanent tooth which was tooth 23 in the presented case. A sharply defined peri-coronal radiolucency around tooth 23 were found at the second control examination. Subsequently, the interval of radiographic examination has been extended to 6 months.

**Fig. 7:**
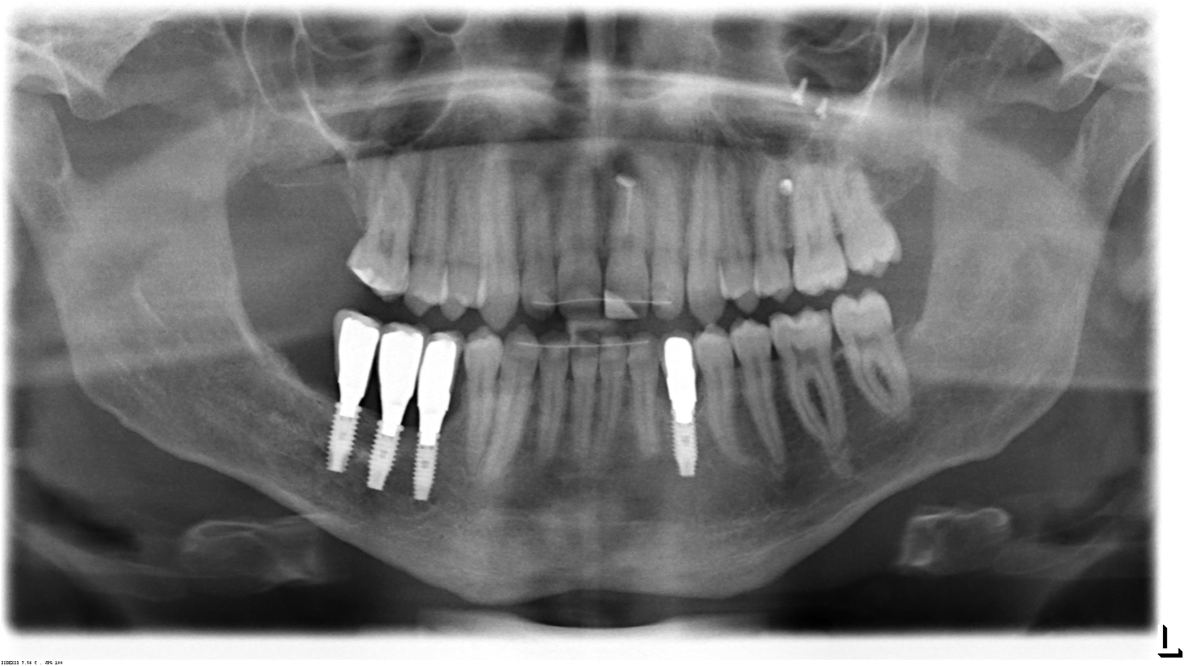
The control OPT at the age of 20 years. A sufficient osseointegration of the implants is obvious 4 years after implant placement. In the left posterior maxilla, the screws and the titanium mesh are visible

At the 6-months follow-up after insertion of the prosthetic restorations, OPT, lateral cephalogram and CBCT showed well osseointegrated implants without any sign of bone loss. No recurrence of the surgically removed KCOTs was observed. However, a new cystic lesion in the posterior part of the right sinus maxillaris was found. Tooth 17 was removed due to the close contact to the cystic lesion without complications. Histologic examination proofed another KCOT. At the 12-months follow-up, a lesion in the basal part of the left sinus maxillaris was observed. As the radiographic control showed a rapid progression of the lesion in the left sinus it was surgically removed under general anesthesia. Due to the strong adherence of the cyst to the bone it had to be resected. The resulting oroantral fistula was closed using a titanium supported membrane (Cytoplast® d-PTFE-Membrane, RIEMSER Dental, Greifswald, Germany). The histologic analysis again revealed a KCOT. Up to the patient’s age of 20 years, the follow-up in semi-annular intervals were without any pathologic finding (Fig. 7).

## Discussion

As shown in the presented case, cystic alterations are frequently diagnosed incidentally. In order to avoid extensive lesions, frequent dental check-ups are recommended during the growth period [14, 33–35]. A treatment including multiple medical disciplines is necessary for the rehabilitation of patients suffering from tumors [33, 36]. On the other hand, the patient’s compliance is inevitable due to the time consuming and stressful therapy. Beginning with the complete removal of the keratocystic odontogenic tumors to avoid later recurrence the rehabilitation requires cooperation, mental capability and compliance especially during the period of orthodontic treatment. In case of children or adolescents, extensive information and communication during the treatment period is important. Furthermore, a genetic counseling of the entire family is recommendable due to the dominant inheritance.

Currently, there is sparse literature regarding the treatment and the rehabilitation of adolescents suffering from KCOT [18, 22, 37]. Wilson and Deboni et al. described young patients treated by enucleation of the keratocystic odontogenic tumors and subsequent orthodontic rehabilitation including tooth extraction as part of the treatment plan [22, 37]. Marsupialization of the KCOTs would have been another treatment option with a comparable success rate to enucleation [38]. A disadvantage over enucleation is the considerable extended treatment period which was found to be around 11 months in a recent study [39]. The extended treatment time might be associated with a reduced quality of life and is requiring a high level of compliance of the patient which might not be given in children and adolescents. Furthermore, when multiple cysts are in situ a surgical removal of the KCOTs might be the treatment of choice. However, in the presented case the patient did not want the long-lasting treatment by marsupialization. Curettage and peripheral ostectomy are frequently performed in addition to enucleation. In the presented case, it was not performed for the mandibular KCOTs in order to prevent any lesion of the inferior alveolar nerve. This might be associated with a higher risk of recurrence of the KCOTs [40]. However, in consideration with the impaired quality of life resulting from a lesion of the inferior alveolar nerve and as the KCOTs could be removed in toto it was decided to perform enucleation alone. Currently, no sign of recurrence was present in the mandible. In the posterior left maxilla, the bone had to be resected due to the strong adherence to the cystic lesion. This could be considered as more invasive treatment lowering the risk of recurrence. However, a more conservative surgical procedure would have been desirable as the resection resulted in an oroantral fistula.

Furthermore, the reconstruction of large defects is challenging. Although autogenous bone is considered as the “gold standard” in bone grafting the juvenile body offers limited amounts of autogenous bone which is challenging to graft extended defects. Additionally, the use of autogenous bone might be associated with donor site morbidity [28]. Adolescents, who are physically active might be limited in their daily life. Thus, in the present case, the defect reconstruction was performed using an allogenic graft block. The ideal bone graft is biocompatible, stable and supports bone remodeling. In several case reports, the suitability of allogenic materials to reconstruct osseous defects in adolescents was shown [41–44]. However, the using of bone grafts might be associated with delayed healing [45]. On the other hand, for the patient the use of allogenic graft materials is beneficial because a second surgery to harvest bone from the chin or the hip might be associated with donor site morbidity [46]. If there remains a vertical volume deficit after grafting with allogenic bone a compensation using vertical distraction osteogenesis is possible after cessation of growth [47–50]. In the present case, vertical distraction osteogenesis was performed in the right mandible to achieve a sufficient implant site. For the planned implant sites xenogenic bone graft was used. In clinical studies, xenogenic materials showed promising results considering the rapid osseointegration of dental implants [51–53]. In the presented case, bovine material was used due to promising clinical experiences in our clinic. Furthermore, a lower volume loss was expected by applying Cerabone® [54]. As the graft material served as space holder a larger particle size might be beneficial to allow bone formation.

The placement of dental implant in the adolescent is discussed controversially. The fixed position during the growth of jaws, unpredictability and the disarrangement of the dental arches have been considered as limitations [55]. On the other hand, missing teeth might cause difficulties in speech and mastication as well as impairment in the patient’s social life [56]. In the presented case, the cessation of growth was estimated on the basis of a hand radiograph. This method of skeletal age assessment is considered to be more reliable than the clinical age assessment regarding individual growth development and degree of maturation [31]. However, a wide variety has to be respected. In the presented case, a decision was made to insert dental implants after the estimated cessation of growth as implants placed in maturing adolescents have shown promising long results [56]. However, long-term studies are currently missing. Recently, some cases were reported showing satisfactory results after implant borne prosthetic rehabilitation [47, 57–61]. Isler et al. reported the case of a 22-year old male patient being provided with a ridge prosthesis fixed on four implants after cyst enucleation [61]. Two years following the treatment, there was no sign of recurrence and the implants remained stable. In the case of a 26-year old male patient the surgical therapy included the decompression and enucleation of the KCOT and the surgical removal of the impacted tooth [60]. During the follow-up period of 31 months, Brkic et al. observed no sign of recurrence and the fixed bridge borne on two implants was functional. Castro-Núñez et al. described a case with an implant-supported overdenture inserted in a bone after bone transport distraction following partial maxillectomy [47]. After 9 years of follow-up, no sign of recurrence was obvious. The patient showed a satisfactory full functional occlusal and dental rehabilitation.

For the diagnostic work-up and the planning of the implant position three-dimensional imaging using CBCT has been a valuable tool. The horizontal and vertical dimensions of potential lesions as well as the alveolar bone can be visualized in detail, which is beneficial, especially in cases of reduced bone volume [62].

During the transitional dentition, radiographical check-ups in intervals of 3 months including the developing tooth germs are recommended to identify potential lesions in an early stage [14, 23, 63, 64]. As these recommendations would cause a considerable exposure to radiation in our case the radiographical check-ups in three-months intervals were performed until the eruption of the last permanent tooth. Subsequently, the interval was extended to 6 months. Thus, an early detection of translucent lesions in the regions of teeth 17 and 18 as well as 28 was possible. Subsequently, the extraction of 17 and the surgical removal of the wisdom tooth germs was indicated due to the development of another keratocystic odontogenic tumor. However, a balance between exposure of radiation and the risk of missing the recurrence of a cystic lesion has to be found.

## Conclusion

In the present case, the dental and facial rehabilitation of a juvenile patient suffering from keratocystic odontogenic tumors has shown satisfying results by performing enucleation of the KCOT and simultaneous defect augmentation with an allograft. The allogenic material seems to be suitable for reconstructing osseous defects in adolescent. However, the volume stability has to be considered carefully. If there is a vertical deficit of the allogenic bone blocks it can be compensated by distraction osteogenesis. Orthodontic treatment until the end of growth is required in order to form the dental arch and to maintain space. The oral rehabilitation with dental implants was possible using three-dimensional planning and guided surgery following bone augmentation. The time point of implant insertion depends on dental and skeletal maturity and should be performed only after the termination of the growth. However, a consistent follow-up is recommendable regarding the tendency to relapse. Furthermore, with regard to the risk of the occurrence of basal cell carcinomas frequent dermatological full-body screenings should be obligatory.

## Abbreviations

BCNS: Basal Cell Nevus Syndrome
CAD: Computer-Aided Design
CAM: Computer-Aided Manufacturing
CBCT: Cone Beam Computed Tomography
KCOT: KeratoCystic Odontogenic Tumors
NBCCS: Nevoid Basal Cell Carcinoma Syndrome
OCT: Odontogenic Ceratocyst
OPT: Orthopantomogram
PTCH1: Patched1
PTCH2: Patched2
